# Development and validation of LC–MS/MS with in-source collision-induced dissociation for the quantification of pegcantratinib in human skin tumors

**DOI:** 10.4155/bio-2016-0199

**Published:** 2017-01-23

**Authors:** Monique Zangarini, Neil Rajan, Marina Danilenko, Philip Berry, Silvio Traversa, Gareth J Veal

**Affiliations:** 1 Newcastle Cancer Centre Pharmacology Group, Newcastle University, Newcastle upon Tyne, NE2 4HH, UK; 2 Institute of Genetic Medicine, Newcastle University, Newcastle upon Tyne, NE2 4HH, UK; 3 Creabilis Therapeutics, Colleretto Giacosa, Italy

**Keywords:** LC–MS/MS, pegcantratinib, PEGylation, tropomyosin receptor kinase A, tumor

## Abstract

**Aim:**

Pegcantratinib is a mini-PEGylated K252a derivative, under clinical evaluation as an anticancer agent acting through inhibition of the tropomyosin receptor kinase. A method for quantifying pegcantratinib in skin tumor biopsies of patients was required to determine tumor drug penetration.

**Methods & results:**

A sensitive and PEGylated molecule specific HPLC–MS/MS method coupled with in-source collision-induced dissociation was developed. The method exhibited excellent precision (coefficient of variation ≤8.5%), accuracy in the range 95–102%, high and consistent recovery and no matrix effect. The assay was linear across a range of 1–500 ng/ml, with a limit of quantitation of 2.5 ng/ml.

**Conclusion:**

We have developed and validated a method for analyzing pegcantratinib in human tumor biopsies, with the approach successfully applied to clinical trial samples.

**Figure 1.**
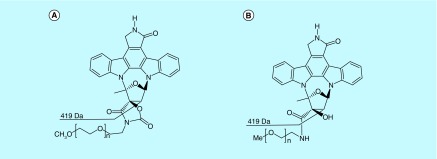
**Chemical structures.** Chemical structures and proposed surrogate ion structures of **(A)** Pegcantratinib and **(B)** CT340.

**Figure 2.**
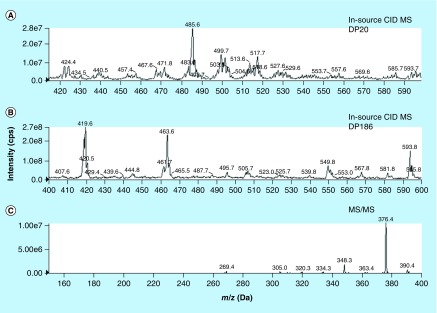
**In-source collision-induced dissociation/Q1 mass spectra of pegcantratinib.** **(A)** at declustering potential 20 V **(B)** at declustering potential 186 V and **(C)** MS/MS mass spectra of pegcantratinib.

**Figure 3.**
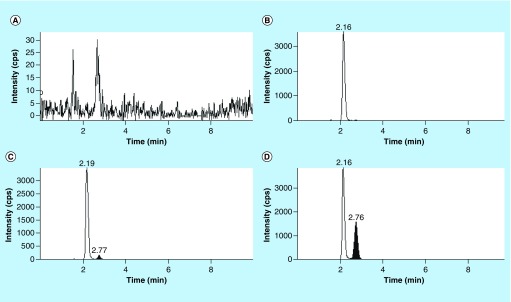
**Typical SRM chromatograms.** **(A)** Extracted blank tumor sample, **(B)** extracted blank tumor sample with internal standard (zero sample), **(C)** extracted tumor sample with pegcantratinib at the LLOQ (2.5 ng/ml) and internal standard **(D)** extracted tumor biopsy sample from a patient treated with pegcantratinib with IS. Note different intensity scale in **(A)**.

**Figure 4.**
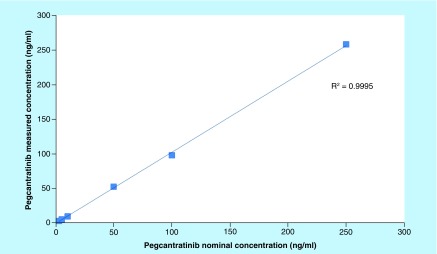
**Calibration curve showing linearity over six concentrations of pegcantratinib (range: 2.5–250 ng/ml) from three separate experiments.**

Pegcantratinib is the first topically applied tropomyosin receptor kinase (TRK) inhibitor that contains mini-PEGylated K252a, which exerts its effects at nanomolar concentrations by inhibiting the intracellular kinase domain of TRK [1]. Unlike other TRK inhibitors, which are available only in oral formulations, the conjugation process of small molecules to PEG gives rise to physicochemical and pharmacological characteristics that optimize these molecules for topical routes of administration. This facilitates the delivery of high levels of the active ingredient to skin tumor cells, while avoiding the systemic side effects of oral applications [2].

Pegcantratinib has recently been evaluated in a Phase llb clinical study setting as a potential treatment in common inflammatory skin conditions, including psoriasis and atopic dermatitis, with a good safety profile observed [3]. The drug is now entering clinical development in patients with germline mutations in the tumor suppressor gene *CYLD*, which include multiple clinical presentations of rare, skin appendage tumors: Brooke–Spiegler syndrome, familial cylindromatosis and multiple familial trichoepitheliomas [4–6]. Patients carrying *CYLD* mutations face repeated disfiguring surgery to control tumor burden, with approximately one in four affected patients undergoing total scalp removal, due to the lack of an available pharmacological treatment [7]. Studies performed in 3D primary cell cultures of cylindroma, treated with salicylic acid and TRK inhibitors, showed them to be particularly sensitive to K252a, with a surviving fraction of 50% (SF_50_) of 366 nM [8].

As part of an ongoing early phase clinical trial, investigations of pegcantratinib tumor penetration and potential correlations between tumor exposure and response are warranted. Therefore, an analytical method was required to quantify pegcantratinib concentrations in tumor samples obtained from patients enrolled on the study. No published assay currently exists to measure pegcantratinib in tumor tissues, with LC–MS/MS method development for PEGylated drugs facing significant challenges due to the heterogeneity and relatively high MW of the PEG fraction. The mass spectrums generated show broad continuous peaks due to the size and charge distributions of PEG that are difficult to interpret and generally not usable for quantitative analysis [9]. However, recent papers have demonstrated that PEGylated therapeutic agents undergo collision-induced dissociation in the ionization source (in-source CID) of the mass spectrometer, generating nonPEGylated precursor ions that can be used for drug quantification [10,11].

We have developed a sensitive and specific HPLC-MS/MS assay, coupled with in-source CID, for direct quantitative measurement of pegcantratinib in human skin tumors. The method requires a small amount of tumor tissue, simple homogenization and extraction and short analysis time. After HPLC separation, the PEGylated small molecule undergoes in-source fragmentation in the instrument ionization source, generating a small molecule pegcantratinib-specific fragment ion that can be used as surrogate for the quantitative analysis. We validated the method according to the European Medicines Agency (EMA) guidance on bioanalytical method validation [12] and demonstrated its applicability to determine the drug's tumor distribution in skin tumor punch biopsies obtained from patients with *CYLD* mutations participating in the current Phase Ib/IIa clinical trial.

## Experimental

### Standards & chemicals

Analytical reference standards of pegcantratinib and CT340, used as internal standard (IS), were provided by Creabilis Therapeutics Srl (Colleretto Giacosa, Italy) and are shown in Figure 1. Analytical grade methanol, acetonitrile, DMSO and formic acid were purchased from Fisher Scientific (Leicester, UK).

### Biological specimens

Human skin tumor biopsies were collected as routinely excised tumor from patients. These samples were snap frozen and stored at -80°C until analysis. Control tumor samples (no drug treatment) were used to prepare the standards and quality control (QC) samples. Biopsies to be analyzed were excised at the end of treatment, subdivided into three different layers (top, middle and bottom), snap frozen and stored at -80°C prior to analysis of pegcantratinib levels.

### Standard & QC solutions

Stock solutions of pegcantratinib for standards and QCs were prepared in DMSO at a concentration of 5 mg/ml. All stock solutions were diluted serially in methanol to generate working solutions with final pegcantratinib concentrations of 25, 50, 100, 500, 1000 and 2500 ng/ml for standards, and 80, 800 and 1600 ng/ml for working solutions of QCs. These working solutions were used to prepare the standard points of the calibration curve and the QC samples in homogenate control tissue, respectively. A stock solution of the IS (CT340) was prepared at 5 mg/ml in DMSO. The IS working solution was prepared at a concentration of 10 μg/ml by diluting the stock solution with methanol. All stock and working solutions were stored at -20 °C prior to use.

### Tissue homogenate, standards & QC samples

Control tumor samples were weighed and homogenized using an Ultra Turrax homogenizer in 1% formic acid (1:5 ratio; w/v) and were used for preparation of calibration curve and QC samples. Calibration curve standards were prepared by adding 10 μl of the working standard solutions to tumor homogenate (90 μl), to produce final concentrations of 2.5, 5.0, 10.0, 50.0, 100 and 250 ng/ml. A blank sample (homogenate processed without IS), a zero blank sample (homogenate processed with IS) and three concentrations of QC samples in triplicate were included alongside the standard curve. QC samples were prepared by adding 10 μl of working QC solutions to blank homogenate tumor (90 μl) to generate concentrations of 8.0, 80.0 and 160 ng/ml.

### Processing samples

Aliquots of homogenate (100 μl) from study samples, standards or QC samples were added with 10 μl (100 ng) of the IS working solution and 900 μl of acetonitrile and samples were vortex-mixed for 20 s. Following extraction, samples were centrifuged at 4000 × *g* for 6 min at 4°C. The supernatants obtained were transferred to Eppendorf polypropylene tubes, evaporated to dryness under nitrogen at 37°C, and then reconstituted in 100 μl of mobile phase (MP) A and MP B in the proportion 1:1, v/v. MP A consisted of 1% formic acid in water and MP B was 1% formic acid in acetonitrile. After vortex mixing, samples were centrifuged at 4000 × *g* for 6 min at 4°C, supernatants were transferred to autosampler glass vials and 10 μl of each sample injected onto the HPLC–MS/MS system.

### Chromatography conditions

The HPLC system consisted of a Series 200 autosampler and micropump (Perkin Elmer, Beaconsfield, Bucks, UK). Samples were separated on a Kinetex C18 chromatographic column (2.6 μm, 50.0 × 4.6 mm) coupled with a precolumn of the same material, both thermostatically controlled at 44°C. The HPLC system was set at a constant flow rate of 0.5 ml/min and run under gradient conditions: step 1 – 50% MP A for 1 min, step 2 – 50% MP A to 15% over 4 min; step 3 – constant 15% MP A for 2 min; step 4 – 15% MP A to initial conditions over 1 min; step 5 – reconditioning for 2 min.

### MS conditions

Detection was obtained using an API 4000 triple quadrupole mass spectrometer SCIEX (CA, USA). Standard solutions (10 μg/ml) of pegcantratinib and IS were infused at a flow rate of 10 μl/min to optimize MS parameters, with mass spectra (MS1) and product ion spectra (MS2) generated in positive ion mode. The HPLC–MS/MS system was equipped with a Turbo Ion Spray source operated at 750°C and voltage of 5500 V. Clinical samples were analyzed with ESI, with zero air as nebulizer gas (60 psi) and heater gas (30 psi). Nitrogen was utilized as curtain gas (20 psi) and collision gas at 4 psi. The declustering potential (DP) was optimized and set to 186 V for in-source CID of both pegcantratinib and CT340. Quantification was carried out in SRM mode with the following transition: *m/z* 419.0→376.4 for both pegcantratinib and IS, following chromatographic separation. Data were processed using the Analyst 1.6.2 software package (SCIEX).

### Method validation

The method validation followed the EMA and the US FDA guidance on bioanalytical method validation [12,13]. The parameters validated were recovery, matrix effect, LLOQ, linearity and range, intra- and inter-day precision and accuracy, carryover effect and stability.

### Recovery

The percentage extraction was determined in triplicate at three QC concentrations (8.0, 80.0 and 160 ng/ml) for pegcantratinib and at 1 μg/ml for the IS in tumor homogenate samples. Absolute recovery was determined by comparing the peak area of pegcantratinib extracted from homogenate tumor samples with the peak area in absence of matrix (true concentration of the analyte in solvent) that represents 100% recovery. The coefficient of variation (CV) was required to be within 15% [13].

### Matrix effect

The matrix effect was assessed in four independent sources of blank matrix for pegcantratinib at the concentration of 100 ng/ml and IS at 10 μg/ml by calculating the matrix factor (MF) for each analyte, that is the ratio of the peak area of the analyte added to a pre-extracted sample to the peak area of the same amount of analyte in solvent. The IS-normalized MF was calculated by dividing the MF of the pegcantratinib by the MF of the IS. The CV of the IS-normalized MF should be within 15% [12].

### Limit of quantification

The LLOQ of the method corresponded to the concentration of the lowest standard with precision ≤20% and accuracy within 80–120% of the nominal value, with a S/N ≥10. The LLOQ was assessed by preparing five samples from a pool of tumor homogenate added with pegcantratinib at the defined concentration [12,13].

### Linearity & range

The calibration curve linearity was validated over three working days, while the linear range was investigated by preparing samples <50% of the LLOQ and >150% of the ULOQ over one working day. For each standard concentration, the ratio of the HPLC-MS/MS peak area for pegcantratinib to IS was calculated and plotted against the nominal concentration of the drug in the sample. Linearity of the standard curves was assessed by regression analysis and the goodness of the fit calculated using Pearson's determination coefficient, R^2^ and by comparing the true and back-calculated concentrations of the calibration standards. The accuracy of back-calculated values of an individual concentration were required to be within 85–115% of the theoretical concentration (80–120% at the LLOQ), and all standards had to meet these criteria [12,13].

### Intra-/interassay precision & accuracy

Five replicates per QC concentration were analyzed for intraday precision and accuracy, while interday precision and accuracy were determined from three separate experiments carried out on different days. The precision of the method at each concentration was reported as the CV value, expressing the SD as a percentage of the mean calculated concentration; accuracy was determined by expressing the mean calculated concentration as a percentage of the nominal concentration. In each run, the concentration measured for QC samples was required to be within 15% of the nominal value [12,13].

### Carryover

Carryover of pegcantratinib and IS were evaluated by placing a blank sample, without analyte or IS, immediately after the highest calibration standard. Response for analyte in the carryover sample was required to be ≤20% of the response at LLOQ. The response for IS in the carryover sample was required to be ≤5% of the response for the control matrix + IS [12,13].

### Stability

The stability of pegcantratinib in homogenate tumor was assessed by analyzing QC samples (low and high concentrations) in triplicate following storage and handling. Short-term stability was assessed in QC samples unextracted and extracted (autosampler stability) from the tumor matrix after 10 days stored at 4°C, the bench-top stability was determined after 4 h at room temperature. Freeze–thaw stability was assessed for three freeze–thaw cycles. We also analyzed the stability of blank frozen homogenate tumor (stored at -20°C), which was used to prepare QCs on the day of the analysis. Long-term stability was assessed in QC samples stored at -20°C for 3 months. The pegcantratinib QC samples were analyzed against a calibration curve, obtained from freshly spiked calibration standards, and the obtained concentrations were compared with the nominal concentrations. The mean concentration at QC concentration was required to be within ±15% of the nominal concentration [12,13].

### Analysis of clinical samples

Biopsies weighing 5–20mg were obtained from cylindroma skin tumors of two CYLD carrier patients enrolled in a Phase Ib open label clinical trial (ISRCTN: 75715723) that received daily topical doses of pegcantratinib (0.5% w/w) for 4 weeks. Biopsies were excised at the end of treatment as described above.

## Results

### Optimization of HPLC–MS/MS parameters & conditions

Using an ESI source in positive ion mode coupled with in-source CID, pegcantratinib and IS generated an abundant fragment ion after applying an optimized DP. As seen previously, the mass spectrum of pegcantratinib at a nondissociating voltage of 20 V (Figure 2A) compared with a high DP of 186 V (Figure 2B), showed the disappearance of the characteristic broad profile associated with PEGylated molecules. This indicates that the polymeric species was dissociated and an abundant new ion species at *m/z* 419.0 was generated (Figure 1). The PEG disassociation was obtained for both the analyte and the IS. The newly obtained ion (*m/z* 419.0) passed through the first quadrupole into the collision cell where the collision energy was optimized to obtain product ions with a high signal (Figure 2C). The characteristic product ions were monitored in the third quadrupole at *m/z* 376.4 (34 eV) and 348.3 (41 eV). Pegcantratinib and IS were quantified using the transitions *m/z* 419.0→376.4 and were distinguished by their different retention times of approximately 2.7 and 2.1 min for pegcantratinib and IS, respectively. Elution of the analytes was rapid and selective. No interfering peaks were present at these retention times, and the peaks were completely resolved from the tumor matrix. Figure 3 presents typical SRM chromatograms: Figure 3A is an extracted blank tumor sample; Figure 3B is an extracted blank tumor sample with IS; Figure 3C is an extracted tumor sample at the LLOQ (2.5 ng/ml) with IS added; and Figure 3D represents an extracted tumor sample from a patient treated with pegcantratinib.

### Recovery

Recovery was evaluated in triplicate over the three QC concentrations by comparing the peak areas of spiked homogenate tumor samples, following extraction, with those obtained from direct injection of pegcantratinib standard solutions in MP. The recovery percentage was >86% for pegcantratinib and 85% for IS. There were no significant variations in CV (<13%) as shown in Table 1.

### Matrix effect

Due to difficulty in obtaining different types of human tumor, the matrix effect was evaluated during the prevalidation study, when four different types of matrix were available.

No matrix effect was observed in four independent matrix sources including three different tumors and normal skin, evaluated by assessment of IS-corrected MF, with a mean ratio of 0.99 ± 0.05 and CV of 5% observed (Table 2).

### Limit of quantification

The LLOQ concentration in tumor homogenate was defined to be 2.5 ng/ml, with precision and accuracy of 6 and 99%, respectively. Single values are shown in Table 3.

### Linearity & range

Examination of linearity over six concentrations of pegcantratinib (range: 2.5–250 ng/ml) yielded a linear correlation of ≥0.995 from three separate experiments (Figure 4). The range of the method was examined between 1.0 and 500 ng/ml over one working day. Results are shown in Table 4.

### Intra-/interassay precision & accuracy

Intra-assay experiments showed precision ranging from 3.6 to 7.3% and accuracy of 95.4–99.0% (n = 5). Interassay experiments carried out over three days showed precision ranging from 4.67 to 8.50% and accuracy of 96.1–99.4% (n = 11) (Tables 5 & 6).

### Carryover & stability

No carryover was detected for pegcantratinib or IS in chromatograms of blank samples injected immediately following ULOQ samples. The stability of pegcantratinib in homogenate tumor was assessed by analyzing QC samples (low and high) in triplicate. Pegcantratinib was stable in tumor for at least 4 h at room temperature and for 10 days at 4°C before and after extraction. Control tumor homogenate, stored at -20°C was shown to be stable for preparation of standards and QCs. Pegcantratinib was stable in tumor homogenate at -20°C over 3 freeze–thaw cycles.

### Clinical trial tumor distribution

The present method was applied to the quantitative analysis of pegcantratinib in human skin tumor biopsies obtained following 4 weeks of daily topical pegcantratinib treatment, from two CYLD carrier patients enrolled in a Phase lb open label clinical trial. The punch biopsies obtained were sliced in three different layers to determine drug penetration. Results presented in Table 7 show a clear trend toward higher drug concentration in the top layers, with drug also able to penetrate to the inner layers.

## Discussion

The TRK inhibitor, pegcantratinib, currently being investigated in early phase clinical trials for the treatment of patients with germline mutations in the tumor suppressor gene *CYLD*, provides several challenges in terms of development of an analytical assay for drug quantification. No assay has been published for this mini-PEGylated agent, with assay development for PEGylated drugs historically facing significant hurdles due to the heterogeneity and high MW of the PEG fraction [9]. In addition, as pegcantratinib is being developed in this clinical situation for topical administration, an assay was required to allow the quantitation of drug levels extracted from tumor material collected by punch biopsies.

We have developed a sensitive and specific HPLC–MS/MS assay, coupled with in-source CID, for direct quantitative measurement of pegcantratinib in human skin tumors. The novel method involves HPLC separation of the PEGylated small molecule, followed by in-source fragmentation in the instrument ionization source, generating a small molecule pegcantratinib-specific fragment ion that can be used as surrogate for the quantitative analysis. Excellent sensitivity was achieved, with an LLOQ below values previously published for PEGylated molecules [10,11]. We have successfully validated the method according to the EMA guidance on bioanalytical method validation [12] and demonstrated its applicability to determine the drug's tumor distribution in skin tumor punch biopsies obtained from patients with CYLD mutations participating in the current clinical trial.

## Conclusion & future perspective

The bioanalytical method described, based on simple tumor homogenization and acetonitrile extraction followed by HPLC–MS/MS coupled with in-source CID analysis, was validated for the quantitative measurement of the PEGylated molecule, pegcantratinib, in human tumor biopsies. The method requires small amounts of tumor matrix; it is selective, sensitive, precise and accurate. It was successfully employed to measure concentrations of pegcantratinib in human skin tumor biopsies of CYLD carrier patients enrolled in a Phase Ib open label clinical trial. The preliminary clinical data obtained showed tumor drug absorption and penetration to inner tumor layers. Data concerning the tumor exposition will provide useful information for investigating correlations between tumor exposure and response in an expanded Phase Ib/IIa clinical trial.

**Table 1.** **Recovery of pegcantratinib from human tumor homogenate at quality control concentrations (n = 5).**

**Pegcantratinib concentration (ng/ml)**	**Recovery (%) ± SD**	**CV (%)**
8.00	89.7 ± 11.9	13.2
80.0	93.7 ± 8.94	9.54
160	86.4 ± 10.4	12.0
Internal standard	84.9 ± 1.61	1.90

CV: Coefficient of variation.

**Table 2.** **Matrix effect of four different types of human tissue homogenate on pegcantratinib (100 ng/ml) and internal standard (1μg/ml).**

**Tissue type**	**Pegcantratinib (MF)**	**CT340 (MF)**	**Ratio**
Skin with anagen follicles	1.05	1.05	1.00
Cylindroma and spiradenoma	0.97	0.96	1.01
Cylindroma only	1.07	1.05	1.02
Spiradenoma only:	0.92	1.01	0.91
– Mean	1.00	1.02	0.99
– SD	0.07	0.04	0.05
– CV (%)	7.1	3.9	5.0

CV: Coefficient of variation; MF: Matrix factor.

**Table 3.** **LLOQ of pegcantratinib in human tumor homogenate.**

**Actual concentration (ng/ml)**	**Found concentration (ng/ml)**	**Accuracy (%)**
2.5	2.22	89
–	2.57	103
–	2.58	103
–	2.41	96
–	2.59	104
Mean (n = 6)	2.47	–
SD	0.16	–
Accuracy (%)	99	–
Precision (%)	6.0	–

**Table 4.** **Interday linearity, accuracy and precision of calibration curves of pegcantratinib in human tumor homogenate.**

**Day**	**Pegcantratinib concentration (ng/ml)**							
	**1.00**	**2.50**	**5.00**	**10.0**	**50.0**	**100**	**250**	**500**
1	0.97	2.63	4.82	9.22	50.3	104	261	481
2	NA	2.59	4.95	9.11	57.0	92.8	267	NA
3	NA	2.46	5.02	10.5	50.3	97.1	247	NA
Mean (n = 4)	NA	2.56	4.93	9.61	52.5	98.0	258	NA
SD	NA	0.09	0.10	0.77	3.87	5.65	10.3	NA
Accuracy (%)	97	102	99	96	105	98	103	96
Precision (%)	NA	3	2	8	7	6	4	NA

NA: Not applicable.

**Table 5.** **Intraday precision and accuracy of the method for the analysis of pegcantratinib in human tumor homogenate.**

	**Nominal concentration (ng/ml)**		
	**8.00**	**80.0**	**160**
Measured concentration	8.47	72.7	154
	7.52	69.6	151
	7.18	82.1	160
	7.27	81.9	163
	8.14	75.4	164
Mean (n = 5)	7.72	76.3	158
SD	0.56	5.56	5.68
Accuracy (%)	96.5	95.4	99.0
Precision (%)	7.3	7.3	3.6

**Table 6.** **Interday precision and accuracy of the method for the analysis of pegcantratinib in human tumor homogenate.**

	**Nominal concentration (ng/ml)**		
	**8.00**	**80.0**	**160**
Measured concentration	8.47	72.7	154
	7.52	69.6	151
Day 1	7.18	82.1	160
	7.27	81.9	163
	8.14	75.4	164
Day 2	9.02	78.2	138
	8.49	77.5	150
	8.85	76.7	154
Day 3	7.88	78.2	162
	7.21	76.5	171
	7.47	77.0	177
Mean (n = 11)	7.95	76.9	159
SD	0.68	3.59	10.74
Accuracy (%)	99.4	96.1	99.1
Precision (%)	8.50	4.67	6.77

**Table 7.** **Pegcantratinib tumor concentrations obtained in three tumor layer levels from two CYLD mutation carrier patients treated in a Phase Ib trial.**

**Patient number**	**Tumor concentration (ng/ml)**		
	**Top layer**	**Middle layer**	**Bottom layer**
1	321	41.1	11.0
2	279	19.5	16.2

**Executive summary**

**Background**

- An LC method coupled with in-source collision-induced dissociation MS was developed and validated to quantify pegcantratinib in skin tumor biopsies of patients enrolled in an early phase clinical to determine tumor penetration.

**Experimental**

- The sample preparation procedure required a 10 mg sample for tissue homogenization and involved protein precipitation with acetonitrile following addition of internal standard.

**Results & discussion**

- The method is selective, precise, accurate and sensitive, with an LLOQ of 2.5 ng/ml in tumor.
- The preliminary clinical data showed tumor drug absorption and penetration to inner tumor layers.
